# GATA6 Exerts Potent Lung Cancer Suppressive Function by Inducing Cell Senescence

**DOI:** 10.3389/fonc.2020.00824

**Published:** 2020-06-12

**Authors:** Wensheng Chen, Zhipeng Chen, Miaomiao Zhang, Yahui Tian, Lu Liu, Ruirui Lan, Guandi Zeng, Xiaolong Fu, Guoqing Ru, Wanting Liu, Liang Chen, Zhenzhen Fan

**Affiliations:** ^1^Key Laboratory of Functional Protein Research of Guangdong Higher Education, Institute of Life and Health Engineering, College of Life Science and Technology, Jinan University, Guangzhou, China; ^2^International Department, The Affiliated High School of SCNU, Guangzhou, China; ^3^Department of Radiation Oncology, Shanghai Chest Hospital, Shanghai Jiaotong University, Shanghai, China; ^4^Department of Pathology, Zhejiang Provincial People's Hospital, People's Hospital of Hangzhou Medical College, Hangzhou, China

**Keywords:** lung cancer, tumor suppressor genes, GATA6, p21, AKT

## Abstract

Lung cancer is the leading cause of cancer-related deaths worldwide. Tumor suppressor genes (TSGs) play a critical role in restricting tumorigenesis and impact the therapeutic effect of various treatments. However, TSGs remain to be systemically determined in lung cancer. Here, we identified GATA6 as a potent lung cancer TSG. GATA6 inhibited lung cancer cell growth *in vitro* and tumorigenesis *in vivo*. Mechanistically, GATA6 upregulated p53 and p21 mRNA while it inhibited AKT activation to stabilize p21 protein, thus inducing lung cancer cell senescence. Furthermore, we showed that ectopic expression of GATA6 led to dramatic slowdown of growth rate of established lung tumor xenograft *in vivo*.

## Introduction

Lung cancer is the leading cause of cancer-related deaths globally (1–3). Non-small cell lung cancer (NSCLC) is the most frequently diagnosed pathological type of lung cancer. Chemotherapy, targeting therapy, and immunotherapy are mainstream treatment options for lung cancer patients in clinic (4, 5). Unfortunately, despite the advances in multimodal therapies, the prognosis for lung cancer patients remains disappointingly dismal, with overall 5-year survival rate of only around 17% (6). The difficulties in developing effective therapies for lung cancer patients stem from our limited understanding of lung tumorigenesis.

Gain-of-function mutations in driver oncogenes and loss-of-function mutations in tumor suppressor genes (TSGs) are thought to coordinately drive transformation of lung epithelial cells into tumorigenic cells. Currently, driver oncogenes are relatively well-studied, with multiple targeting drugs available for lung cancer patients in clinic. However, TSGs remain to be systemically determined in lung cancer (7, 8).

GATA6 is a typical member of GATA transcription factor family, consisting of GATA1–6. GATA family members play a critical role during embryonic development through their complex involvements in cell fate decisions and tissue morphogenesis (9, 10). GATA6, like other members, binds consensus sequence (A/T)GATA(A/G) (11, 12). Strong expressions in cardiac tissue and endodermal derivatives have been reported for GATA6 (13, 14). Direct knockout of GATA6 results in embryonic lethality, rendering it difficult to delineate its role during early development (15).

GATA6 has been reported to play an important role in maintaining the function of respiratory tract by inducing Muc5b (16). GATA6 has also ambiguously been reported to be involved in lung cancer development, with both oncogenic and tumor suppressive roles reported. Ma et al. showed that GATA6 conferred drug resistance for NSCLC cells by inducing autophagy (17). However, Li et al. reported that GATA6 inhibited lung cancer cell migration and invasion (18). Moreover, while GATA6 has been implicated in transdifferentiation of NSCLC into liver cancer phenotype (19), Zito's report suggested that GATA6 can enhance differentiation of lung cancer cells to NSCLC (20). Until now, it remains an open and critical question to determine the exact role of GATA6 in the process of lung cancer development.

Here we clarify a tumor suppressive role for GATA6 in NSCLC. We show that expression level of GATA6 is higher in para-tumoral tissues than in lung cancer tissues and is significantly associated with better prognosis of NSCLC patients. Ectopic expression of GATA6 inhibits proliferation of NSCLC cells, colony formation in 2-D plates, and soft-agar culture. Ectopic expression of GATA6 in lung epithelial compartment inhibits lung cancer development. Mechanistically, GATA6 overexpression results in senescence of lung cancer cells through accumulation of p53 and p21 and concurrent downregulation of AKT activity. Furthermore, we showed that ectopic expression of GATA6 resulted in a significant slowdown of growth rate of lung tumor xenografts.

## Results

### GATA6 Is a Potent and Clinically Relevant TSG in Lung Cancer

**Figure 1:**
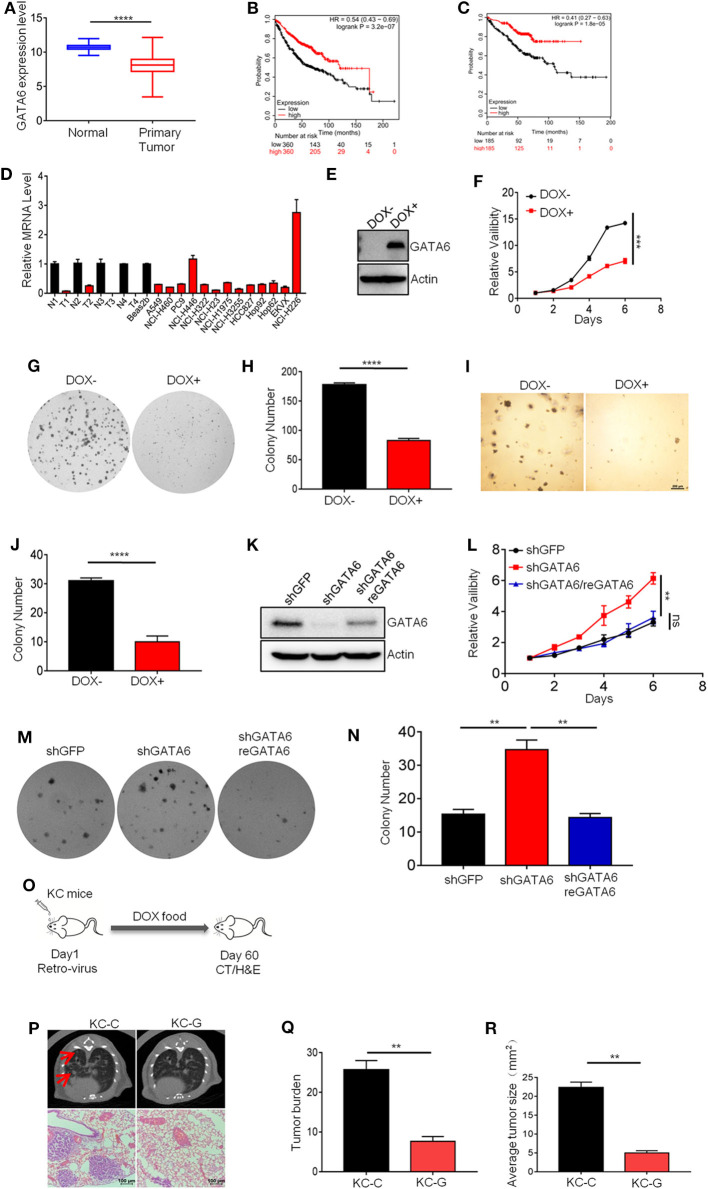
GATA6 is a potent and clinically relevant tumor suppressor gene in lung cancer. **(A)** GATA6 mRNA expression in lung cancer tissue and para-tumoral tissue among the lung cancer patients from TCGA (http://xena.ucsc.edu). **(B)** K–M survival of lung cancer patient (all stage, *n* = 360) (http://kmplot.com). **(C)** K–M survival of lung cancer patient (Stage I, *n* = 185) (http://kmplot.com). **(D)** qRT-PCR analysis of GATA6 expression in lung cancer cell lines along with clinical samples. N, paratumor tumoral tissue; T, tumor. **(E)** Western blot analysis of doxycycline-inducible GATA6 expression in stable cell lines of A549i. **(F)** The CCK8 assay of proliferation of stable cell lines of A549i treated with or without DOX (DOX+, DOX–). **(G)** Representative images of colony-forming assay of A549i in the presence or absence of DOX (DOX+, DOX–). **(H)** Statistics of colony number in **(G)**. **(I)** Soft-agar colony-forming assay of A549i in the presence or absence of DOX (DOX+, DOX–). **(J)** Statistics of soft-agar colony result shown in I (*n* = 3 per group). **(K)** Western blot analysis of GATA6 expression in NCI-H226 cells transfected with shRNA targeting GATA6 mRNA and rescued by overexpression of shRNA-resistant cDNA. **(L)** The CCK8 assay of proliferation of engineered NCI-H226 cells. Cells were transfected with shRNA targeting GATA6 mRNA or re-expression GATA6. **(M)** Representative images of colony-forming assay of NCI-H226. Cells were transfected with shRNA targeting GATA6 mRNA or re-expression GATA6. **(N)** Statistics of result represented in **(M)**. **(O–R)** Inhibition of development of mutant Kras-driven lung cancer by GATA6 in transgenic mouse model. **(O)** Schematics of intranasal instillation of lentivirus for overexpressing GATA6 in DOX inducible TetO-Kras^G12D^/CC10rtTA mice model (referred to as KC). **(P)** Tumor burdens recorded through computed tomography (CT) scanning for TetO-Kras^G12D^/CC10rtTA mice (upper panel). Red arrow-head highlighted the tumors. Hematoxylin and eosin staining of lung section of TetO-Kras^G12D^/CC10rtTA mice (lower panel). **(Q,R)** Statistics of the tumor burden and tumor size of **(P)**. Data are representative of three independent experiments and were analyzed by unpaired *t*-test. Error bars denote SEM. **P < 0.01; ***P < 0.001; and ****P < 0.0001.

Our earlier study has revealed the tumor suppressive function of a GATA transcription family member (21). We therefore checked the functional impact of GATA6 on lung cancer development. Analysis of TCGA data revealed significantly lower GATA6 mRNA level in primary lung tumor in comparison to para-tumoral tissues (Figure 1A). Moreover, higher expression level of GATA6 in lung adenocarcinoma was significantly correlated with longer survival of patients in all stages (Figure 1B). Of note, this trend was also found in stage I patients (Figure 1C), strongly suggesting GATA6 as a clinically relevant TSG.

In order to validate its TSG function, we sought to ectopically express GATA6 in lung cell lines with lower baseline expression and knockdown in those with relatively higher expression. For this purpose, we checked GATA6 expression level in lung cancer cell lines commonly used in cancer research community through quantitative reverse transcription PCR (qRT-PCR), including four pairs of lung adenocarcinoma/para-tumoral tissues as references for GATA6 expression in tumoral and normal lung tissues. We found comparable GATA6 mRNA level between tumor samples and cancer cell lines, which was consistently lower than that of para-tumoral tissues (Figure 1D). Among the lung cancer cell lines, NCI-H446 (small cell lung cancer cell line) and NCI-H226 (lung squamous cell carcinoma cell line) expressed relatively higher level of GATA6, while the rest were relatively lower (Figure 1D). We then generated doxycycline (DOX)-inducible expression of GATA6 for A549 cells (lung adenocarcinoma cell line), a cell line with low expression of GATA6 (referred to as A549i) (Figure 1E) and found that ectopic expression of GATA6 significantly slowed down the growth rate and inhibited the ability of A549i to form colonies in 2-D plates (Figures 1F–H). We also found that DOX treatment inhibited the ability of A549i to form colonies in soft agar plate (Figures 1I,J). Conversely, we knock down GATA6 expression in NCI-H226, a cell line with relatively higher level of expression. Interestingly, we found that GATA6 knockdown significantly promoted growth rate and enhanced colony-forming ability of NCI-H226, which was rescued by re-expression of shRNA resistant GATA6 (Figures 1K–N). Taken together, these data strongly argued that GATA6 was a potent TSG in lung cancer.

Having confirmed its tumor suppressor function *in vitro*, we went on to confirm the tumor suppressor function of GATA6 *in vivo* using transgenic lung cancer mouse model. We generated TetO-**K**RAS^G12D^/**C**C10rtTA bitransgenic mice (referred to as KC mice) following our earlier protocol (22). These mice developed lung adenocarcinoma after being fed with doxycycline (DOX)-containing diet for 2 months. We then asked whether forced expression of GATA6 inhibited lung cancer development. For this purpose, we intranasally delivered lentiviruses for overexpressing GATA6 or control virus into KC mice and then fed them with doxycycline-containing diet for 2 months (Figure 1O and Supplementary Figure 1). Computed tomography (CT) analysis revealed significantly lower tumor burdens in lungs of GATA6-overexpressing KC mice (referred to as KC-G) than in Control KC mice (referred to as KC-C) (Figure 1P). Examination of hematoxylin and eosin (H&E)-stained lung tissue sections revealed lung adenocarcinomas with features of diffused bronchial adenocarcinomas in lungs of KC-C mice (Figure 1P). We found that around 4% of these tumors are stage IV adenocarcinoma. In stark contrast, KC-G mice looked largely normal at this stage, with significantly fewer and smaller tumor nodules (Figures 1Q,R). These results convincingly showed that GATA6 was a functional TSG *in vivo*.

### Ectopic Expression of GATA6 Induces Senescence of Lung Cancer Cells

**Figure 2:**
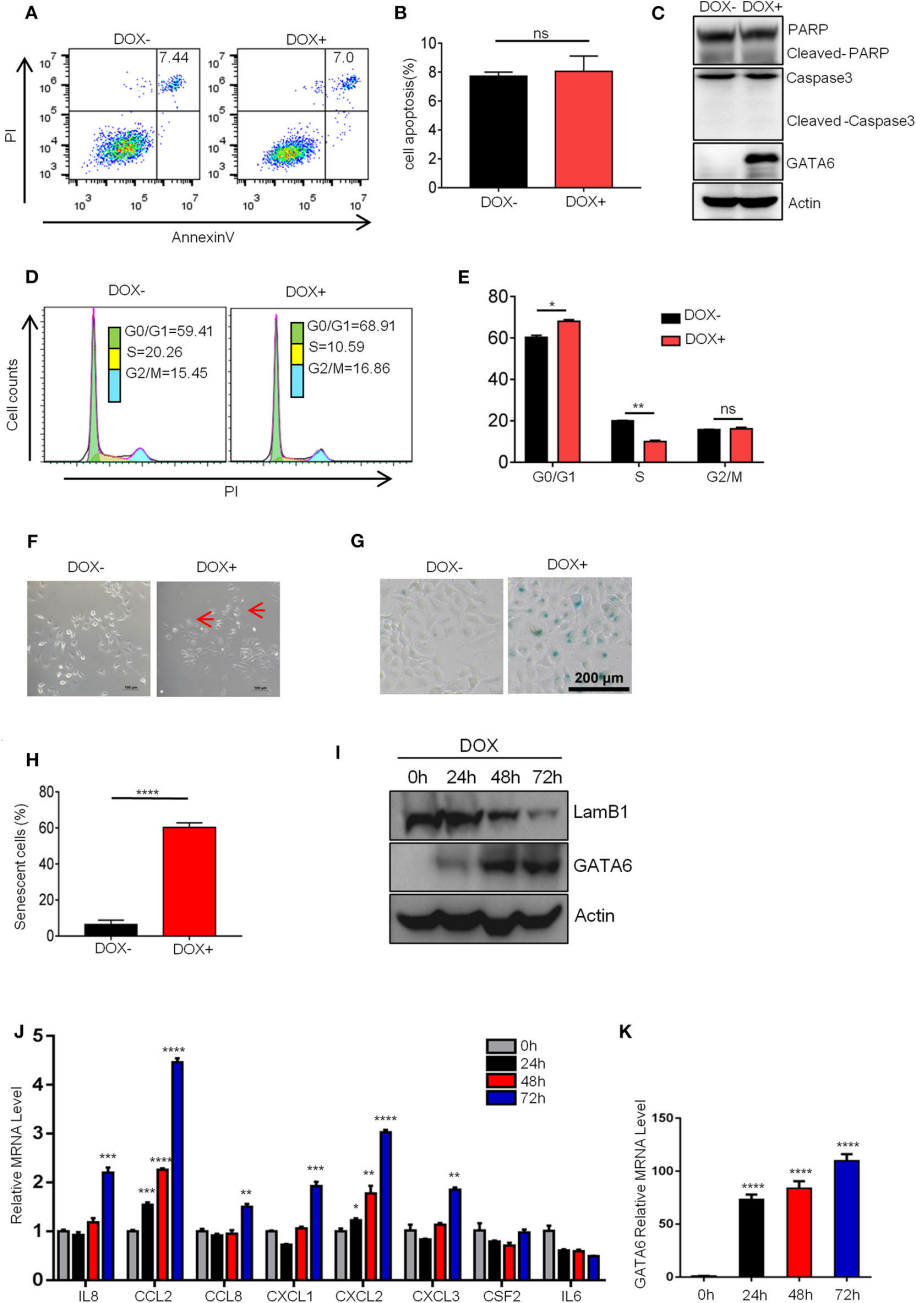
GATA6 overexpression induces senescence of lung cancer cells. **(A,B)** FACS analysis of annexin-V and propidium iodide (PI)-stained apoptotic cells following DOX treatment. The A549i cells (1 × 10^5^) were treated with or without DOX (2 μg/ml) for 48 h before FACS analysis. **(A)** Representative image of Annexin-V-FITC/PI staining. **(B)** Quantitative data of Annexin-V-FITC/PI staining. **(C)** Western blot analysis of caspase 3 and PARP cleavage in A549i cells treated with or without DOX. **(D,E)** FACS analysis of propidium iodide (PI)-stained cells. A549i cells were treated with or without DOX for 48 h and stained with propidium iodide. DNA content was analyzed by flow cytometry. Results are represented as percent of cell population in G0/G1, S, and G2/M phases of the cell cycle. **(D)** Representative image. **(E)** Statistics of **(D)**. **(F)** Morphology of A549i cells expressing GATA6. A549 cells were treated with or without DOX for 48 h (scale bars, 100 μm). Red arrow-head highlighted the senescent cells. **(G,H)** Senescence-associated β-galactosidase staining. A549i cells were treated with or without DOX for 48 h. **(G)** Representative staining. **(H)** Statistics of the positive percentage of senescence cells. **(I)** Western blot analysis of nuclear LaminB1 expression in A549i cells treated with or without DOX. A549i (5 × 10^4^) cells were seeded in six-well plates and cells were treated with DOX (2 μg/ml). Cells were harvested at 24, 48, and 72 h after DOX treatment and subjected to Western blot analysis for LamB1 expression. **(J)** qRT-PCR analysis of mRNA level of SASP markers in GATA6 expressing A549i cells at indicated time points. **(K)** qRT-PCR validation of GATA6 expression in the A549i cells at indicated time points. Data are representative of three independent experiments and were analyzed by unpaired *t*-test. Error bars denote SEM. *P < 0.05; **P < 0.01; ***P < 0.001; and ****P < 0.0001.

We next investigated how GATA6 influenced lung cancer cell growth. TSGs inhibit tumor formation mainly by inducing cell-cycle arrest, apoptosis, and/or senescence. FACS analysis revealed no significant difference in PI and Annexin V double-positive population of A549i before and after DOX treatment (Figures 2A,B), suggesting that apoptosis was not involved in TSG function of GATA6 in lung cancer. Consistently, we found that A549i exhibited no significant alterations in cleavage of PARP1 and caspase 3 in response to DOX treatment (Figure 2C).

We then checked cell-cycle distribution of A549i in response to GATA6 expression. Strikingly, GATA6 expression led to a significant decrease of S phage and a concurrent increase of G0/G1 in A549i populations (Figures 2D,E). Cell senescence, a stable exit of cell cycle, is a frequent effect in tumor cells caused by TSG expression. We then checked senescence of A549i cells in response to GATA6 expression. We found that A549i cell exhibited enlarged, flattened, and irregular shape after DOX treatment, a typical morphological feature of senescent cell (Figure 2F). Interestingly, we were able to confirm GATA6-induced senescence of A549 through β-galactosidase staining (Figures 2G,H). Moreover, Western blot analysis revealed that GATA6 expression led to downregulation of LaminB1 in nuclei, a senescence-associated biomarker in A549 [(23, 24); Figure 2I]. We also check markers of senescence-associated secretory phenotype (SASP) (25) and found that ectopic expression of GATA6 increased the mRNA level of IL8, CCL2, CCL8, CXCL1, CXCL2, and CXCL3 (Figures 2J,K). Taken together, these data showed that GATA6 exerted TSG function by inducing senescence of lung cancer cells.

Earlier, Gao et al. reported that TGF-β2-Wnt-7b signaling axis mediated GATA4-induced senescence in lung cancer cells (21). We asked whether GATA6 exerted its effect through a similar mechanism. To this end, we checked mRNA expression level of *TGFB2* and *WNT7B* in DOX-treated A549i cells. qRT-PCR showed that overexpression of GATA6 diminished expression level of *TGFB2* and *WNT7B* in DOX-treated A549i cells (Supplementary Figures 2A,B). However, overexpression of neither *TGFB2* nor *WNT7B* rescued cellular senescence of A549i cells induced by GATA6 overexpression (Supplementary Figures 2C–F). Moreover, administration of recombinant TGF-β protein failed to rescue the senescence in DOX-treated A549i (Supplementary Figures 2G,H).

Taken together, our data showed that GATA6 exerted its TSG function by inducing senescence of NSCLC cells through a mechanism different from that used by other members of the GATA family.

### GATA6 Induces Senescence Through Upregulating p21 Protein Level

**Figure 3:**
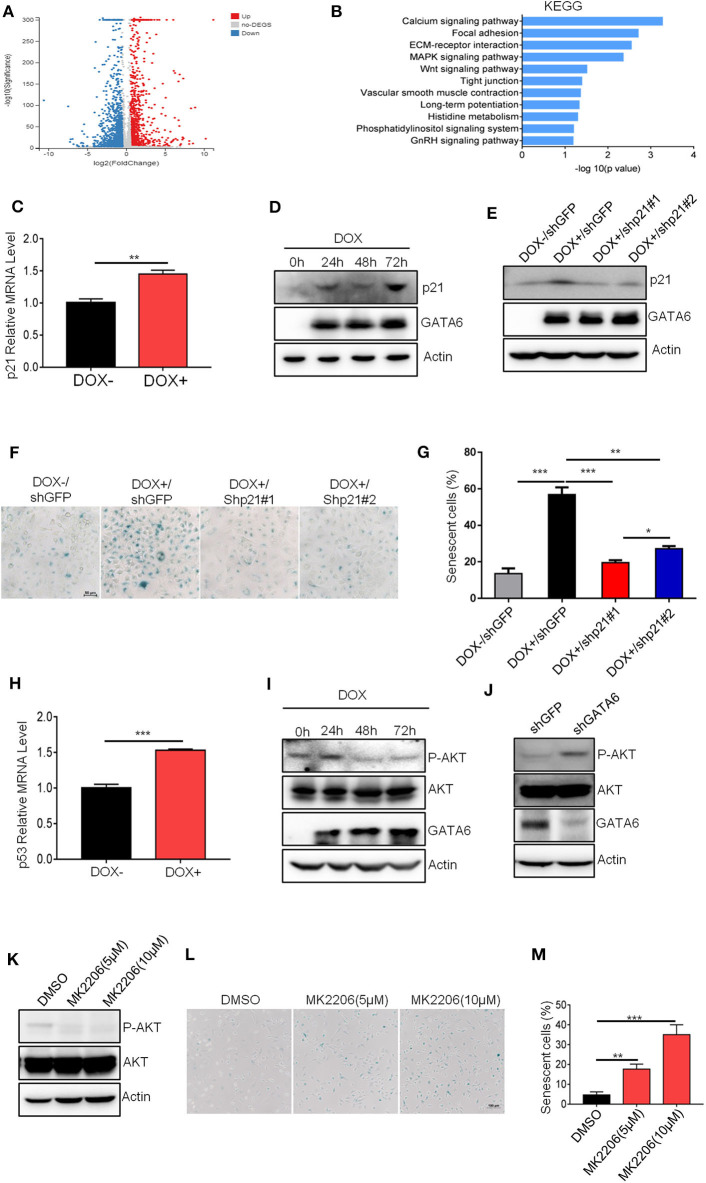
GATA6 induces senescence through upregulating p21 protein level. **(A)** Volcano plot displaying differentially expressed genes between before and after GATA6 expression. **(B)** KEGG analysis of genes upregulated in GATA6 overexpressing A549 cells. **(C)** qRT-PCR analysis of p21 genes mRNA level in GATA6 expressing A549i cell lines. **(D)** Western blot analysis of p21 expression in GATA6 expressing A549i cell lines at the indicated time points. A549i (5 × 10^4^) cells were seeded in six-well plates and cells were treated with or without DOX (2 μg/ml). Cells were harvested at 24, 48, and 72 h and analyzed through Western blot. **(E)** Western blot analysis of p21 expression in DOX (2 μg/ml)-treated A549i cells after transfected with shp21#1 or shp21#2. A549i (5 × 10^4^) cells seeded in six-well plates and transfected with shp21#1 or shp21#2. Twenty-four hours after transfection, cells were treated with DOX for 48 h and were harvested for immunoblot analysis with indicated antibodies. **(F**,**G)** Senescence-associated β-galactosidase staining. A549i cells were treated with or without DOX (2 μg/ml) after transfected with shp21#1 or shp21#2 for 48 h. **(F)** Representative staining. **(G)** Statistics of the positive percentage of senescence cells. **(H)** qRT-PCR analysis of p53 genes mRNA level in GATA6 expressing A549i cell lines. **(I)** Representative Western blot showing the levels of total and phosphorylated AKT in the lysates of A549i cells. A549i cells were treated with or without DOX (2 μg/ml) for 24, 48, and 72 h; whole-cell lysates from the indicated cells were analyzed by immunoblots with the indicated antibodies. **(J)** Representative Western blot showing the levels of total and phosphorylated AKT in the lysates of NCI-H226 cells. NCI-H226 cells were transfected with shGFP or shGATA6 for 48 h; whole-cell lysates from the indicated cells were analyzed by immunoblots with the indicated antibodies. **(K)** MK-2206 inhibited AKT phosphorylation. A549 cells were treated with MK-2206 for 48 h; whole-cell lysates from the indicated cells were analyzed by immunoblots with the indicated antibodies. **(L,M)** Cell senescence in A549 cells treated with MK-2206. A549 cells were treated with MK-2206 for 48 h. β-galactosidase staining was conducted on A549 cells. **(L)** Representative staining. **(M)** Statistics of the positive percentage of senescence cells. Data are representative of three independent experiments, and were analyzed by unpaired *t*-test. Error bars denote SEM. *P < 0.05; **P < 0.01; and ***P < 0.001.

We next investigated the molecular mechanisms underlying the GATA6-induced senescence in lung cancer cell line. We checked the alteration of gene expression at transcriptomic level through RNA-sequencing of A549i with or without DOX treatment (GEO accession number: GSE147447). GATA6 overexpression resulted in 547 genes upregulated and 710 genes downregulated for more than two-fold (Figure 3A). Genes upregulated in A549i cells were analyzed by KEGG. We found pathways regulating calcium signaling, focal adhesion, ECM–receptor interaction, MAPK signaling, etc. as the most significantly altered by DOX treatment in A549i cells. However, pathways regulating senescence were not highlighted (Figure 3B).

Since GATA6 inhibited tumor formation by inducing cell-cycle arrest and senescence, we manually checked expression level of genes that were reported to be involved in regulation of cell cycle and found that *CDKN1A* (encoding p21), a potent cell-cycle inhibitor and senescence inducer, was upregulated (Figure 3C and Supplementary Figure 3A). P21 binds cyclin D-CDK4 complex to inhibit its function, thus preventing phosphorylation of downstream effector cell-cycle mediators to halt cell cycle (26). Western blot analysis confirmed higher protein level of p21 in DOX-treated A549i cells (Figure 3D). To validate if p21 is a critical protein mediating GATA6-induced senescence in A549i cells, we knocked down p21 using shRNAs in A549i cells and found that DOX treatment resulted in significantly lower number of senescent cells in response to p21 downregulation (Figures 3E–G). Earlier reports demonstrated that p53 was an upstream transcription factor of p21 expression (27). In line with this, we found that p53 was upregulated in response to GATA6 overexpression (Figure 3H and Supplementary Figure 3B). Previous studies have also demonstrated that inhibition of AKT function and hence blocking p21 phosphorylation at T145 is critical for p21 to function as a cell senescence inducer (28, 29). We then checked AKT activity and found that GATA6 overexpression inhibited AKT activity as reflected by diminished S473 phosphorylation in DOX-treated A549i cells (Figure 3I). Conversely, GATA6 knockdown significantly promoted phosphorylation of AKT in NCI-H226 cells (Figure 3J). Critically, administration of MK-2206, a potent AKT inhibitor, drastically upregulated senescence in A549 cells in a dose-dependent manner (Figures 3K–M). Hence, DOX treatment diminished phosphorylation of T145 of p21 (Supplementary Figure 3C). Taken together, our data strongly argued such a scenario: GATA6 expression resulted in higher p53 and p21 mRNA and p21 proteins. Meanwhile, GATA6 inhibited AKT activity, thus rendering T145 of p21 unphosphorylated, which allow p21 to potently induce cell senescence.

### Ectopic Overexpression of GATA6 Significantly Inhibits Growth of Established Tumor Nodule *in vivo*

**Figure 4:**
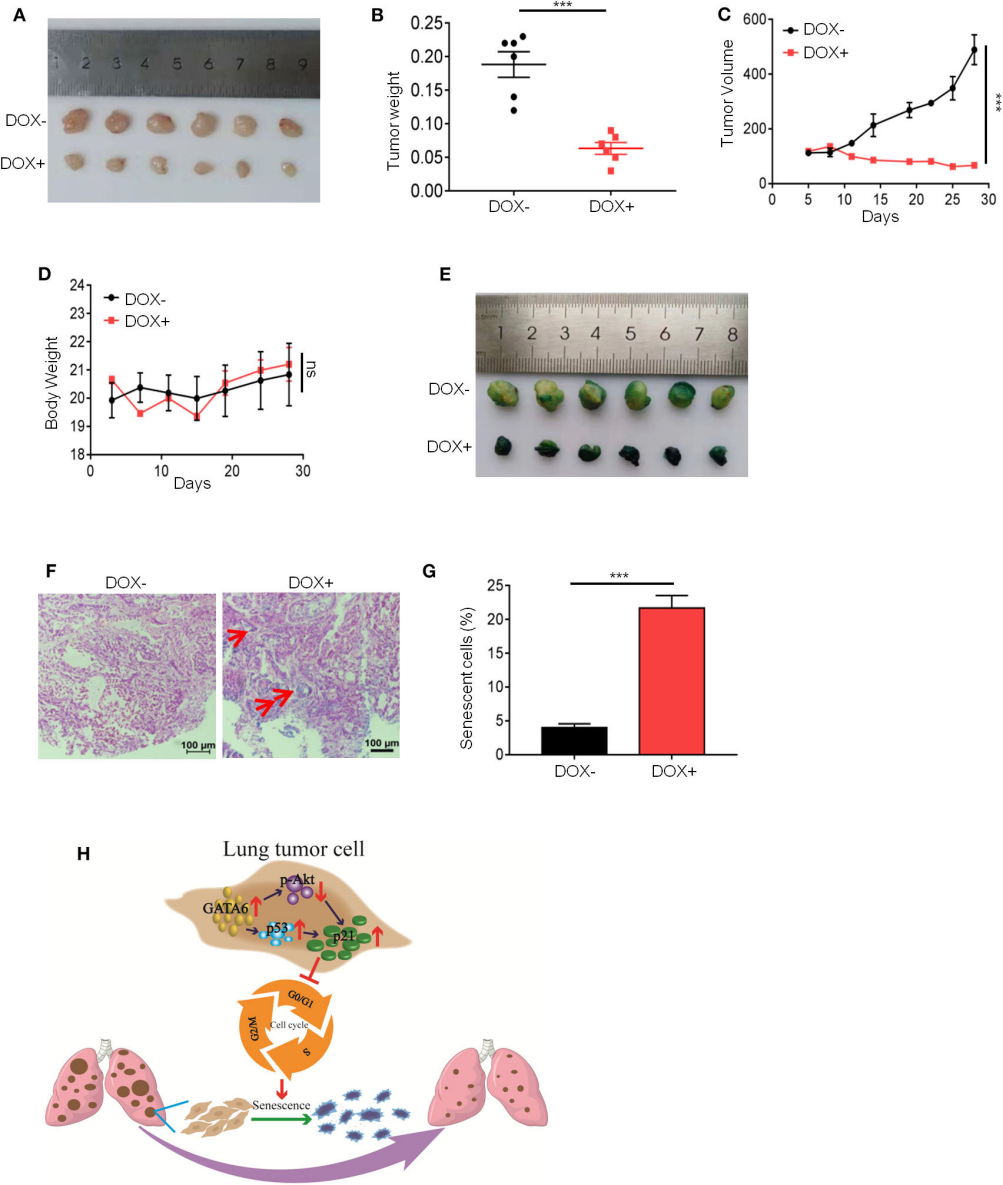
Ectopic expression of GATA6 induces senescence of lung tumor. **(A)** The inhibitory effect of GATA6 overexpression on growth of xenografted tumors. Nude mice were inoculated with A549i cells and treated with DOX-containing (*n* = 4) or control diet (*n* = 4) for 28 days when tumor volume reached around 100 mm^3^. Representative images of the tumors harvested by the end of experiment were shown. **(B)** Tumor weight on day 28 (*n* = 4) mentioned in **(A)**. **(C)** Growth curve of tumor volume was graphed against day post-DOX treatment. Tumor volume was recorded every 4 days by measuring its diameter with Vernier caliper in the mice detailed in A [tumor volume (mm^3^) = *D* × *d*^2^/2, where *D* is the longest and *d* is the shortest diameter, respectively]. **(D)** Body weight of tumor-bearing mice. Body weight was recorded every 4 days in the mice detailed in **(A)**. **(E)** Cell senescence in xenografted tumors overexpressing GATA6. Mice were treated with DOX-containing or control diet and β-galactosidase staining was conducted. **(F,G)** H&E staining of β-galactosidase-stained tumor nodules. **(F)** Representative image. Red arrow-head highlighted the senescent cells. **(G)** Statistics of the positive percentage of senescence cells of **(F)**. **(H)** Model of how GATA6 regulates cellular senescence and tumor growth. Data are representative of three independent experiments, and were analyzed by unpaired *t*-test. Error bars denote SEM. ***P < 0.001.

Our *in vitro* data (Figures 1–3) suggested a therapeutic opportunity for GATA6-deficient lung cancer patients: restoring GATA6 in tumor cells may be a treatment option. Given those highly efficient systems under development or in clinical trial for delivering genes of interest to targeted tumor tissues (30), our hypothesis may be of paramount translational importance. We then went on to test the effect of GATA6 expression on established tumors. We inoculated A549i cells subcutaneously in nude mice and randomized them for treatment with DOX-containing or control diet when tumors reached a volume of around 100 mm^3^. Interestingly, while tumors in control-diet-fed mice continued growing, significant slowdown of tumor growth was seen in the DOX-treated group (Figure 4A). On day 28 post-treatment, we found that the average volume and weight of tumors in DOX-treated group was significantly lower than those in the control-treated group (Figures 4B,C). Of note, DOX treatment was not toxic as indicated by constant weight of mice during the experiment (Figure 4D).

In line with our *in vitro* findings, IHC staining revealed that DOX treatment resulted in overexpression of GATA6 in tumor cells of xenograft. We also detected upregulation of p21 and p53 mRNA level through qRT-PCR and downregulation of p-AKT through Western blot analysis in the tumor nodule (Supplementary Figures 4A–E). Consistently, we found robust β-galactosidase staining signals in the DOX-treated group and largely negative in the control group (Figures 4E–G). Taken together, our data suggested that targeted overexpression of GATA6 in tumor cells of GATA6-deficient lung cancers may elicit P53–P21 signaling axis to induce senescence in lung cancer cells.

## Discussion

Recent years have witnessed an intensive research interest in identifying TSGs of lung cancer. In our current study, we found that GATA6 was a clinically relevant TSG in lung cancer. GATA6 overexpression led to senescence of lung cancer cells. We delineated the pathway underlining the tumor-suppressive function of GATA6 for lung cancer cells. Moreover, we confirmed the *in vivo* TSG function of GATA6 and confirmed that restoration of GATA6 expression slows down the growth of established xenografted tumor nodules deficient in GATA6 function.

Real and colleagues recently reported an elegant work showing the tumor-suppressive function of GATA6 in pancreatic cancer (31). Moreover, their subsequent work revealed that GATA6 regulated EMT and tumor dissemination of pancreatic cancer (32). GATA6 has earlier been reported to exert its TSG function by restricting transdifferentiation of lung cancer cells (33). The focus of that study is not on GATA6's impact on cell growth, but on metastasis. However, we found that the most striking effect of GATA6 expression was on growth of lung cancer cells. A similar effect was observed on GATA4, another member of this family (21). Indeed, we confirmed the growth inhibitory effect of GATA6 not only *in vitro* but also *in vivo* using a xenograft tumor model and a transgenic mouse model of autochthonous lung cancer. Due to the limitation of this study, we cannot exclude the possibility that GATA6 impacted tumor growth through modulating differentiation of tumor cells. Whether changes in pathways related to cell adhesion may lead to senescence through indirectly regulating p21 expression remains an open question.

In our study, we revealed an intriguing mechanism underlying the cell senescence-inducing effect of GATA6 through regulating the function of p21. P21 bound to various cyclin–CDK complexes to inhibit the activity of these complexes, thus resulting in a block in cell-cycle progression (34). Reports have also shown that p21 bound to PCNA to inhibit DNA polymerase δ and ε activities (35, 36). However, p21 has also been reported as a positive regulator of cell cycle by promoting assembly and activation of cyclin D1-CDK4/6 complexes (26, 37). Phosphorylation of T145 by AKT transforms p21 from a cell-cycle inhibitor to a cell-cycle promoter (29). GATA6 reinforces the cell-cycle inhibitory function of p21 through simultaneously upregulating the expression and enhancing the function of p21: GATA6 induced the mRNA level of p21; meanwhile, GATA6 expression inhibited AKT activity. Inhibition of AKT activity not only stabilized p21 protein level but also unleashed its cell-cycle inhibitory function on lung cancer cells (Figure 4H). However, the exact mechanism underlying GATA6's negative regulation of AKT activity and positive regulation of p53/p21 expression warrants further study. GATA6 may directly bind to AKT and inhibit its phosphorylation. It could also be that GATA6 inhibited expression of PDK1 or TRAF6, a E3 ligase essential ubiquitination of AKT to promote its phosphorylation (38).

Our *in vivo* data showed that ectopic expression of GATA6 in established xenografted lung tumor nodules led to a significant growth inhibition. This is of paramount translational significance. Currently, several highly efficient systems are under development or in clinical trial to deliver gene of interest into target tumor cells (39). Restoration of GATA6 expression may therefore represent an opportunity for those GATA6-deficient lung cancer patients. Our analysis of TCGA data showed that a significant portion of lung cancer samples were GATA6-low. We could expect this GATA6-low population to benefit from GATA6 restoring therapy if further study confirms it to be safe and effective.

## Materials and Methods

### Plasmid

The pLVX-TetOne-Puro plasmid was purchased from Clontech. The following shRNAs were retrieved from Sigma Mission shRNA Library: shGATA6 (TRCN0000005391) and shGFP (SHC004). psPAX2 and pMD2.G were purchased from Addgene. FLAG-tagged GATA6 was constructed by standard molecular biology techniques.

Shp21#1, shp21#2 were constructed in pLKO-vector (Addgene). The primers are as follows:

shp21#1-Forward: 5′-CCGGCGCTCTACATCTTCTGCCTTACTCGAGTAAGGCAGAAGATGTAGAGCGTTTTTG-3′,

shp21#1-Reverse: 5′-AATTCAAAAACGCTCTACATCTTCTGCCTTACTCGAGTAAGGCAGAAGATGTAGAGCG-3′,

shp21#2-Forward: 5′-CCGGGACAGATTTCTACCACTCCAACTCGAGTTGGAGTGGTAGAAATCTGTCTTTTTG-3′,

shp21#2-Reverse: 5′-AATTCAAAAAGACAGATTTCTACCACTCCAACTCGAGTTGGAGTGGTAGAAATCTGTC-3′.

### Reagents and Antibodies

Protease and phosphatase inhibitor cocktail (Biotech Roche), Trizol reagent (Invitrogen), Lipofectamine 3000 (Invitrogen), Western blotting substrate (Millipore), Cell Counting Kit-8 (CCK8, Dojindo Molecular Technologies), Cell Signaling Senescence β-Galactosidase Staining Kit (cell signaling technology, #9860), Antibody against GATA6 (cell signaling technology, #5851), Antibody against p21 (cell signaling technology, #2947), Antibody against P-p21 (T145) (abcam, ab47300), Antibody against FLAG (cell signaling technology, #14793), and Antibody against β-actin (Sigma, A5316) were used in this study.

### Cell Culture and Generation of Engineered Cell Lines

A549, NCI-H1975, NCI-H3255, NCI-H322, NCI-H460, NCI-H827, NCI-H226, NCI-H23, EKVX, Hop62, Hop92, Beas-2b, PC-9, and HEK293T cells were purchased from ATCC (American Typical Culture Collection). A549 is wild type for TP53 (https://web.expasy.org/cellosaurus/) and Rb (40) and harboring a deletion in CDKN2A (41). NCI-H226 is wild type for TP53, a mutant for CDKN2A (42), and harbors a deletion mutation in Rb (43). All cell lines were maintained in standard tissue culture incubators at 37°C and 5% CO_2_. All lung cancer cells were cultured in RPMI-1640 supplemented with 10% fetal bovine serum (FBS, Gibco, 42G9274K) and 1% penicillin/streptomycin/glutamine (Gibco, 15140122). HEK293T cells were cultured in DMEM supplemented with 10% FBS and 1% penicillin/streptomycin/glutamine.

Generation of engineered cell lines: co-transfecting pLVX-TetOne-Puro-GATA6-FLAG or pLKO-shGATA6 or pLKO-shp21 or pLKO-shGFP, psPAX2, and pMD2.G into HEK293T cells using transfection reagent VigoFect (Vigorous Biotechnology). The culture was replaced with fresh media 6–8 h after the transfection. The supernatant was harvested at 48 h and sterilized through a 0.45-μm filter. The recombinant virus stock was stored at −80°C until use. Cells were infected by the recombinant virus in the presence of 8 μg/ml polybrene (sigma) and were selected with 1 μg/ml puromycin (sigma) for 1 week.

### Cell Proliferation Assay

A549i (1 × 10^3^) cells or NCI-H226 (1 × 10^3^) cells were seeded in 96-well plates and supplemented with DOX (2 μg/ml) for 1, 3, and 5 days. Cell proliferation activity was measured using CCK8 cell counting kit according to the manufacturer's protocol.

### Soft-Agar Colony Formation Assay

Soft-agar colony formation assay was performed in soft agar (0.5% base gel and 0.35% top gel) in six-well plates. Add 1 ml of 0.5% base gel to each well of six-well plates and set aside for 5 min. A549i (1000 cells) cells were mixed with the top gel and DOX (2 μg/ml) and then seeded in six-well plates. 0.5 ml 1 × fluid media containing DOX (2 μg/ml) was added to the surface of the top gel every week. After 3–4 weeks, colonies were stained with 0.05% crystal violet for more than 1 h. Colonies were counted with a dissecting microscope.

### Colony Formation Assay

Cells (1 × 10^3^) were plated in six-well plates and grown for 14 days with the indicated treatment. Then, cells were fixed with 4% formaldehyde in 1 × PBS and stained with 0.05% crystal violet for more than 1 h. Colonies were counted with a dissecting microscope with which diameters larger than 200 μm were counted.

### Senescence Associated β-Galactosidase (SA-β-Gal) Staining Assay

SA-β-Gal staining was performed using the Senescence β-Galactosidase Staining Kit (cell signaling technology, #9860). Briefly, cells (2 × 10^5^) were seeded in six-well plates and cultured until the time of staining. For DOX treatment, DOX (2 μg/ml)-containing medium was changed every other day. After 48 h, cells were stained for SA-β-Gal activity. Blue positive cells from at least 10 randomly selected fields were counted with a dissecting microscope.

### Reverse-Transcription PCR

A549i cells were treated with DOX for 48 h. Total RNA was extracted with Trizol and subjected to quantitative reverse transcription PCR (qRT-PCR) using gene specific primers:

P21-Forward: 5′-AGGTGGACCTGGAGACTCTCAG-3′,

P21-Reverse: 5′-TCCTCTTGGAGAAGATCAGCCG-3′,

P27-Forward: 5′-ATAAGGAAGCGACCTGCAACCG-3′,

P27-Reverse: 5′-TTCTTGGGCGTCTGCTCCACAG-3′,

P53-Forward: 5′-CCTCAGCATCTTATCCGAGTGG-3′,

P53-Reverse: 5′-TGGATGGTGGTACAGTCAGAGC-3′,

CDK1-Forward: 5′-GGAAACCAGGAAGCCTAGCATC-3′,

CDK1-Reverse: 5′-GGATGATTCAGTGCCATTTTGCC-3′,

CDK2-Forward: 5′-ATGGATGCCTCTGCTCTCACTG-3′,

CDK2-Reverse: 5′-CCCGATGAGAATGGCAGAAAGC-3′,

CDK4-Forward: 5′-CCATCAGCACAGTTCGTGAGGT-3′,

CDK4-Reverse: 5′-TCAGTTCGGGATGTGGCACAGA-3′,

Cyclin A-Forward: 5′-CTCTACACAGTCACGGGACAAAG-3′,

Cyclin A-Reverse: 5′-CTGTGGTGCTTTGAGGTAGGTC-3′,

Cyclin E-Forward: 5′-TGTGTCCTGGATGTTGACTGCC-3′,

Cyclin E-Reverse: 5′-CTCTATGTCGCACCACTGATACC-3′,

MDM2-Forward: 5′-TGTTTGGCGTGCCAAGCTTCTC-3′,

MDM2-Reverse: 5′-CACAGATGTACCTGAGTCCGATG-3′.

### SASP (Senescence-Associated Secretory Phenotype) Response

A549i cells were treated with DOX (2 μg/ml) for 24, 48, and 72 h. Total RNA was extracted with Trizol and subjected to quantitative reverse transcription PCR (qRT-PCR) using gene specific primers:

IL-8-Forward: 5′-GAGAGTGATTGAGAGTGGACCAC-3′

IL-8-Reverse: 5′-CACAACCCTCTGCACCCAGTTT-3′

CCL2-Forward: 5′-AGAATCACCAGCAGCAAGTGTCC-3′

CCL2-Reverse: 5′-TCCTGAACCCACTTCTGCTTGG-3′

CCL8-Forward: 5′-TATCCAGAGGCTGGAGAGCTAC-3′

CCL8- Reverse: 5′-TGGAATCCCTGACCCATCTCTC-3′

CXCL1-Forward: 5′-AGCTTGCCTCAATCCTGCATCC-3′

CXCL1-Reverse: 5′-TCCTTCAGGAACAGCCACCAGT-3′

CXCL2-Forward: 5′-GGCAGAAAGCTTGTCTCAACCC-3′

CXCL2- Reverse: 5′-CTCCTTCAGGAACAGCCACCAA-3′

CXCL3-Forward: 5′-TTCACCTCAAGAACATCCAAAGTG-3′

CXCL3-Reverse: 5′-TTCTTCCCATTCTTGAGTGTGGC-3′

CSF2-Forward: 5′-GGAGCATGTGAATGCCATCCAG-3′

CSF2-Reverse: 5′-CTGGAGGTCAAACATTTCTGAGAT-3′

IL-6-Forward: 5′-AGACAGCCACTCACCTCTTCAG-3′

IL-6- Reverse: 5′-TTCTGCCAGTGCCTCTTTGCTG-3′.

### RNA Sequencing

A549i cells were treated with or without DOX for 48 h. Total RNA was isolated from A549i cells using Trizol reagent. RNA sequencing and RNA libraries were performed by the Beijing Genomics Institute (BGI).

### *In vivo* Xenograft Model

Eight-week-old male BALB/c nude mice (Guangdong Medical Lab Animal Center) were used for *in vivo* animal experiments. For xenograft study, mice were inoculated subcutaneously into the right-back with A549i (5 × 10^6^) cells in Matrigel. When tumors reached a volume of around 100 mm^3^, the mice were randomly grouped and fed with either DOX-containing or control diet. The body weight and tumor volume [= *D* × *d*^2^/2 (mm^3^), where *D* is the longest and *d* is the shortest diameter] were monitored every 4 days. At the end of treatment, mice tumors were collected, photographed, and weighed.

### Mouse Treatment

All mice were housed in a pathogen-free environment in Jinan University, and all experimental protocols were approved by the Institutional Committee for Animal Care and Use at Jinan University.

Mouse lines of TetO-Kras^G12D^/CC10rtTA bitransgenic mice were kept in the lab. The TetO-Kras^G12D^/CC10rtTA bitransgenic mice developed lung adenocarcinoma after being fed with doxycycline-containing diet for 2 months. Following this protocol, we intranasally delivered lentivirus overexpression GATA6-FLAG or control virus into TetO-Kras^G12D^/CC10rtTA mice and fed with doxycycline-containing diet for 2 months. Mice were sacrificed and lungs were collected for hematoxylin & eosin (H&E) staining. For quantification of tumor burden, we calculated the total size (mm^2^) of all the tumor regions in H&E sections under a microscope. For the quantification of tumor nodules, we counted the number of visible tumor nodules in the whole lung through computed tomography (CT, PINGSENG Healthcare).

### Statistical Analysis

Statistics were performed using GraphPad Prism 7.04. Data are representative of three independent experiments and were analyzed by unpaired *t*-test Error bars denote SEM. ^*^P < 0.05; ^**^P < 0.01; ^***^P < 0.001; and ^****^P < 0.0001.

## Data Availability Statement

The raw data supporting the conclusion of this article will be provided as Supplementary Files. Otherwise, we will make them available without any undue reservation to any qualified researchers.

## Ethics Statement

This animal study was reviewed and approved by Institutional Committee for Animal Care and Use at Jinan University. Written informed consent was obtained from the owners for the participation of their animals in this study.

## Author Contributions

ZF, WC, ZC, and MZ performed most of the experiments and analyzed the data. ZF, LC, and ZC contributed to the discussion. WC, MZ, ZC, LL, GZ, and YT performed animal preparation and data collection. XF analysed the animal data. ZF, LC, WL, and GR conceived and designed this work and wrote the manuscript. RL participated in some cell experiments during the revision. All authors contributed to the article and approved the submitted version.

## Conflict of Interest

The authors declare that the research was conducted in the absence of any commercial or financial relationships that could be construed as a potential conflict of interest.

## Supplementary Material

Supplementary Figure 1 Immunohistochemical analysis of the expression of GATA6 in DOX- inducible TetO-Kras^G12D^/CC10rtTA mice model. Lentiviruses for overexpressing GATA6-FLAG or control vector were intranasally delivered into Kras^G12D^/CC10rtTA mice and fed with doxycycline-containing diet for 2 months. Mice were sacrificed and lungs were stained with FLAG-antibody. Tumor cells with heavy nuclear staining of GATA6 were highlighted with arrow heads.

Supplementary Figure 2 **(A,B)** qRT-PCR analysis of *TGFB2* and *WNT7B* expression in A549i treated with or without DOX (2 μg/ml). **(C,D)** qRT-PCR analysis of *TGFB2* and *WNT7B* expression in A549i cells after transfected with *TGFB2* and *WNT7B* for 48 h. **(E,F)** Senescence-associated β-galactosidase staining. A549i cells were treated with DOX (2 μg/ml) and transfected with *TGFB2* and *WNT7B* for 48 h. **(E)** Representative staining, **(F)** statistics of the positive percentage of senescence cells. **(G,H)** Failure of rescuing the senescence in DOX treated A549i by recombinant TGF-β protein. A549i (5 × 10^4^) cells were seeded in six-well plates and treated with DOX in the presence or absence of recombinant TGF-β protein for 48 h. Cells were subjected to galactosidase staining. Senescence-associated β-galactosidase were quantified by percentage of cells positive for staining. **(G)** Representative staining. **(H)** Statistics of percentage of senescence cells. Data are representative of three independent experiments, and were analyzed by unpaired *t*-test Error bars denote SEM. ^*^*P* < 0.05; ^**^*P* < 0.01; and ^****^*P* < 0.0001.

Supplementary Figure 3 **(A)** qRT-PCR analysis of mRNA level of cell cycle-related genes in GATA6 expressing A549i cell lines. **(B)** qRT-PCR analysis of p53 or p21 mRNA level in A549 cells after treated with cisplatin. A549 (5 × 10^4^) cells seeded in six-well plates and treated with cisplatin (5 μM) for 48 h. Cells were subjected to qRT-PCR. **(C)** Representative western blot showing the levels of total and phosphorylated p21 in the lysates of A549i cells. A549i (5 × 10^4^) cells were seeded in six-well Plates. Cells were harvested at 48 h after DOX (2 μg/ml) treatment and analyzed through Western blot for P-p21 (T145) and p21 expression. Data are representative of three independent experiments, and were analyzed by unpaired *t*-test Error bars denote SEM. ^*^*P* < 0.05 and ^****^*P* < 0.0001.

Supplementary Figure 4 **(A)** Nude mice were inoculated with 5 × 10^6^ A549i cells (harboring DOX inducible expression of GATA6-FLAG), and treated with DOX-containing or control diet for 28 days when tumors reached a volume of 100 mm^3^. Tumor xenografts were harvested and stained with FLAG-antibody. Tumor cells with heavy nuclear staining of GATA6 were highlighted with arrow heads. **(B)** qRT-PCR analysis of GATA6 mRNA level in xenografted tumors. **(C)** qRT-PCR analysis of p53 mRNA level in xenografted tumors. **(D)** qRT-PCR analysis of p21 mRNA level in xenografted tumors. **(E)** Western blot analysis of P-AKT, AKT and GATA6 expression in xenografted tumors. Data are representative of three independent experiments, and were analyzed by unpaired *t*-test Error bars denote SEM. ^*^*P* < 0.05 and ^**^*P* < 0.01.
