# Supplementary figures and images for: GATA6 Exerts Potent Lung Cancer Suppressive Function by Inducing Cell Senescence
Source: Front Oncol. 2020 Jun 12;10:824. doi: 10.3389/fonc.2020.00824 (PMC7304445; doi:10.3389/fonc.2020.00824)

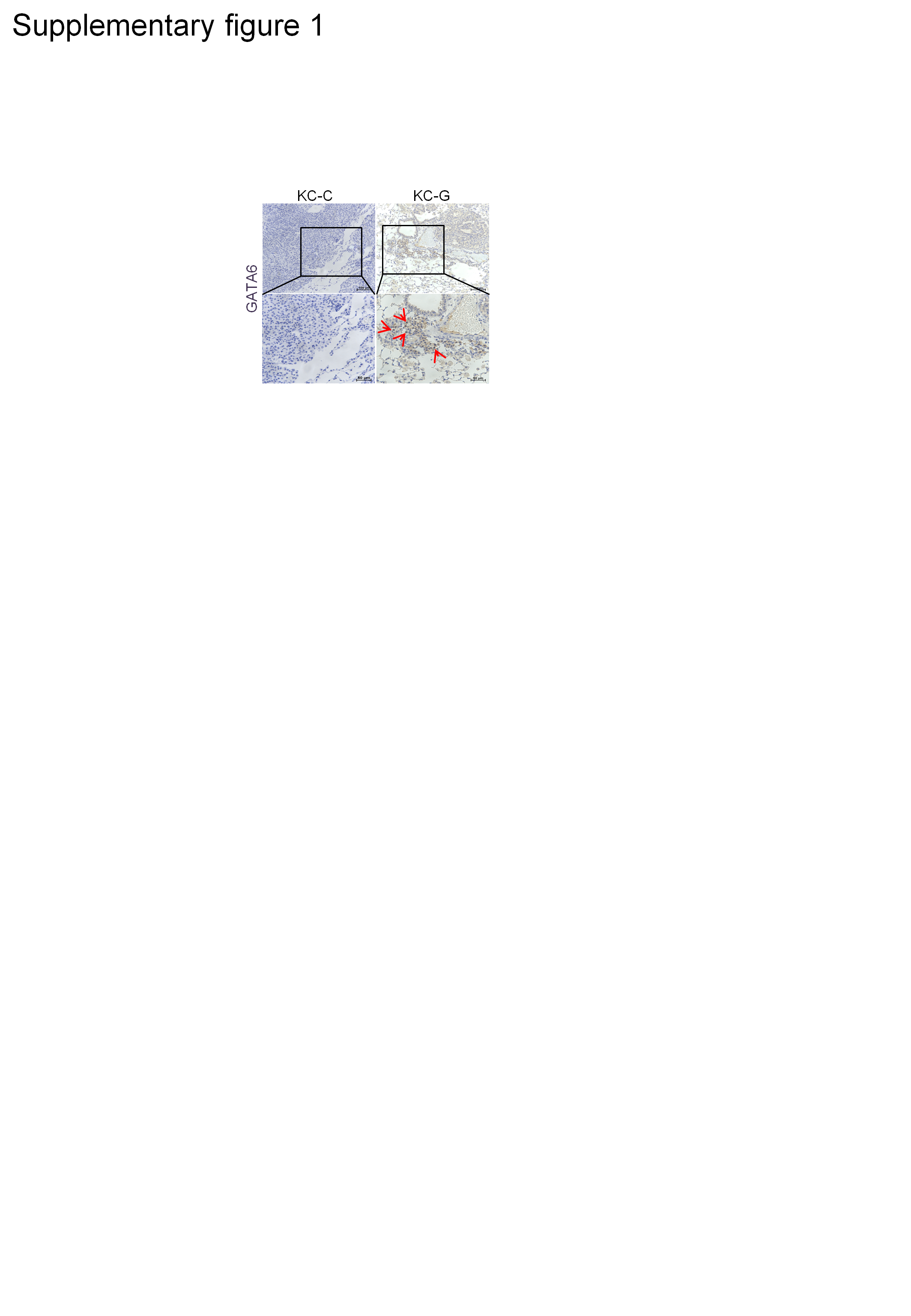

Supplement: Supplementary Figure 1 — Immunohistochemical analysis of the expression of GATA6 in DOX- inducible TetO-KrasG12D/CC10rtTA mice model. Lentiviruses for overexpressing GATA6-FLAG or control vector were intranasally delivered into KrasG12D/CC10rtTA mice and fed with doxycycline-containing diet for 2 months. Mice were sacrificed and lungs were stained with FLAG-antibody. Tumor cells with heavy nuclear staining of GATA6 were highlighted with arrow heads. [file Image_1.TIF]

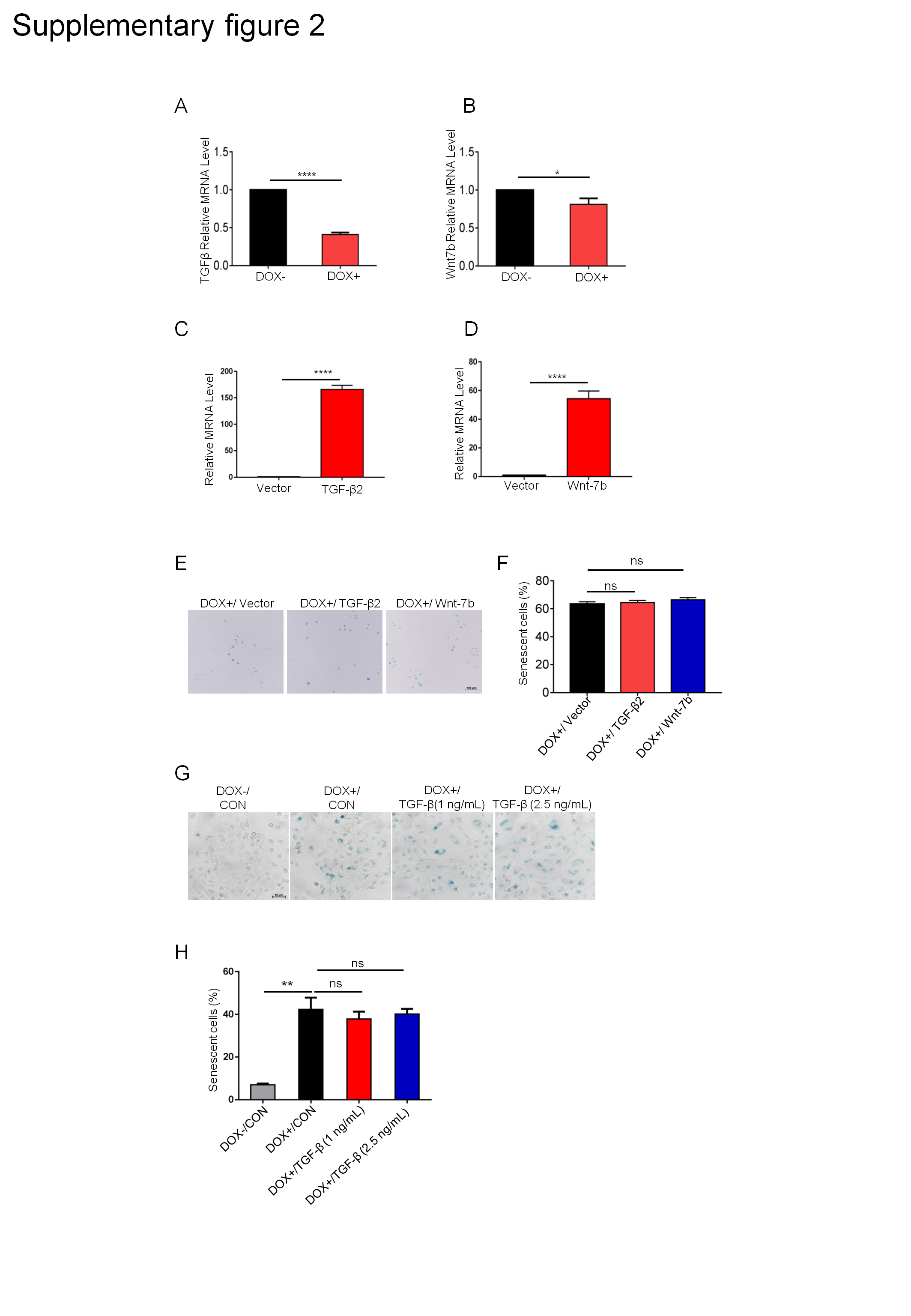

Supplement: Supplementary Figure 2 — (A,B) qRT-PCR analysis of TGFB2 and WNT7B expression in A549i treated with or without DOX (2 μg/ml). (C,D) qRT-PCR analysis of TGFB2 and WNT7B expression in A549i cells after transfected with TGFB2 and WNT7B for 48 h. (E,F) Senescence-associated β-galactosidase staining. A549i cells were treated with DOX (2 μg/ml) and transfected with TGFB2 and WNT7B for 48 h. (E) Representative staining, (F) statistics of the positive percentage of senescence cells. (G,H) Failure of rescuing the senescence in DOX treated A549i by recombinant TGF-β protein. A549i (5 × 10^4^) cells were seeded in six-well plates and treated with DOX in the presence or absence of recombinant TGF-β protein for 48 h. Cells were subjected to galactosidase staining. Senescence-associated β-galactosidase were quantified by percentage of cells positive for staining. (G) Representative staining. (H) Statistics of percentage of senescence cells. Data are representative of three independent experiments, and were analyzed by unpaired t-test Error bars denote SEM. *P < 0.05; **P < 0.01; and ****P < 0.0001. [file Image_2.TIF]

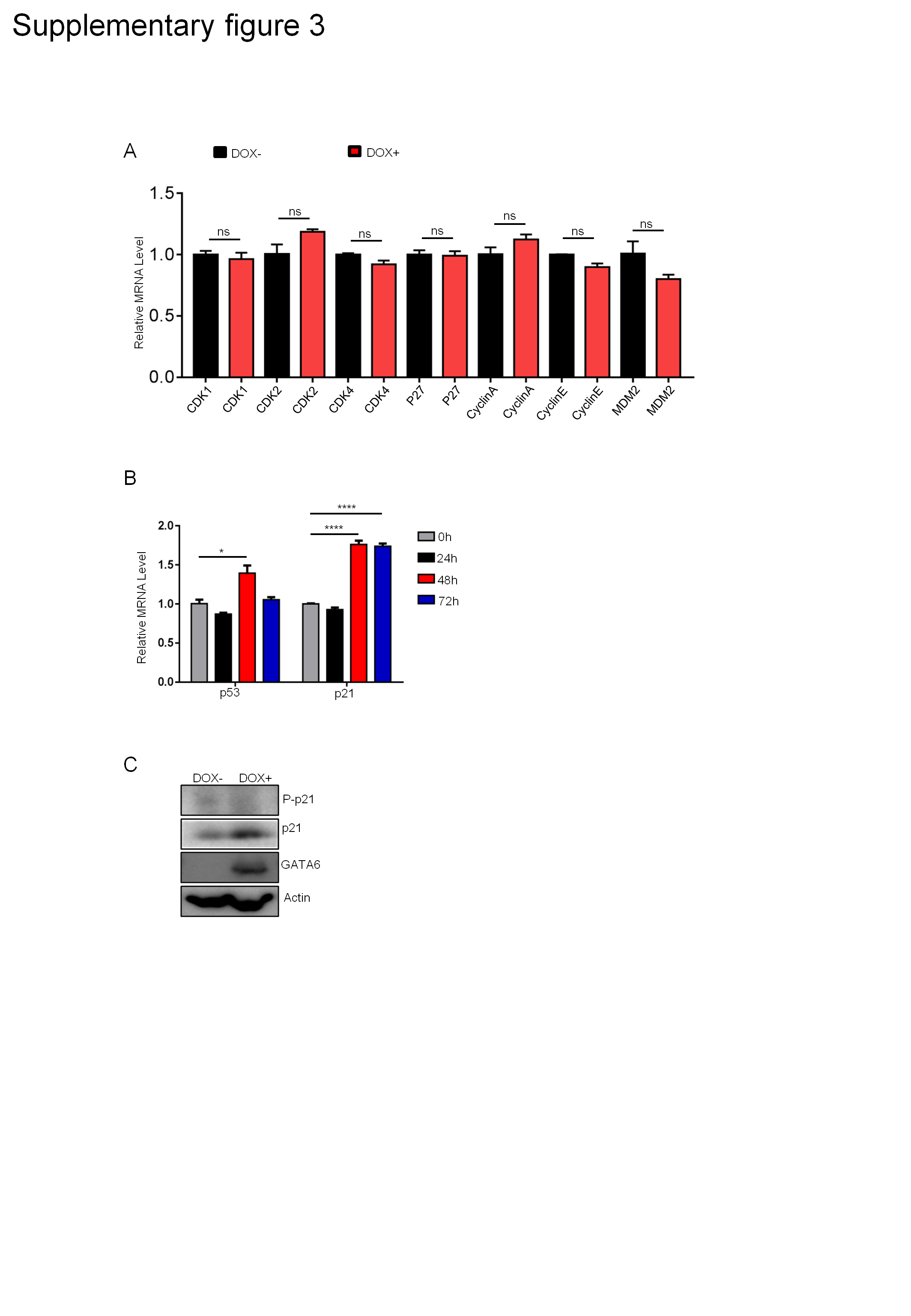

Supplement: Supplementary Figure 3 — (A) qRT-PCR analysis of mRNA level of cell cycle-related genes in GATA6 expressing A549i cell lines. (B) qRT-PCR analysis of p53 or p21 mRNA level in A549 cells after treated with cisplatin. A549 (5 × 10^4^) cells seeded in six-well plates and treated with cisplatin (5 μM) for 48 h. Cells were subjected to qRT-PCR. (C) Representative western blot showing the levels of total and phosphorylated p21 in the lysates of A549i cells. A549i (5 × 10^4^) cells were seeded in six-well Plates. Cells were harvested at 48 h after DOX (2 μg/ml) treatment and analyzed through Western blot for P-p21 (T145) and p21 expression. Data are representative of three independent experiments, and were analyzed by unpaired t-test Error bars denote SEM. *P < 0.05 and ****P < 0.0001. [file Image_3.TIF]

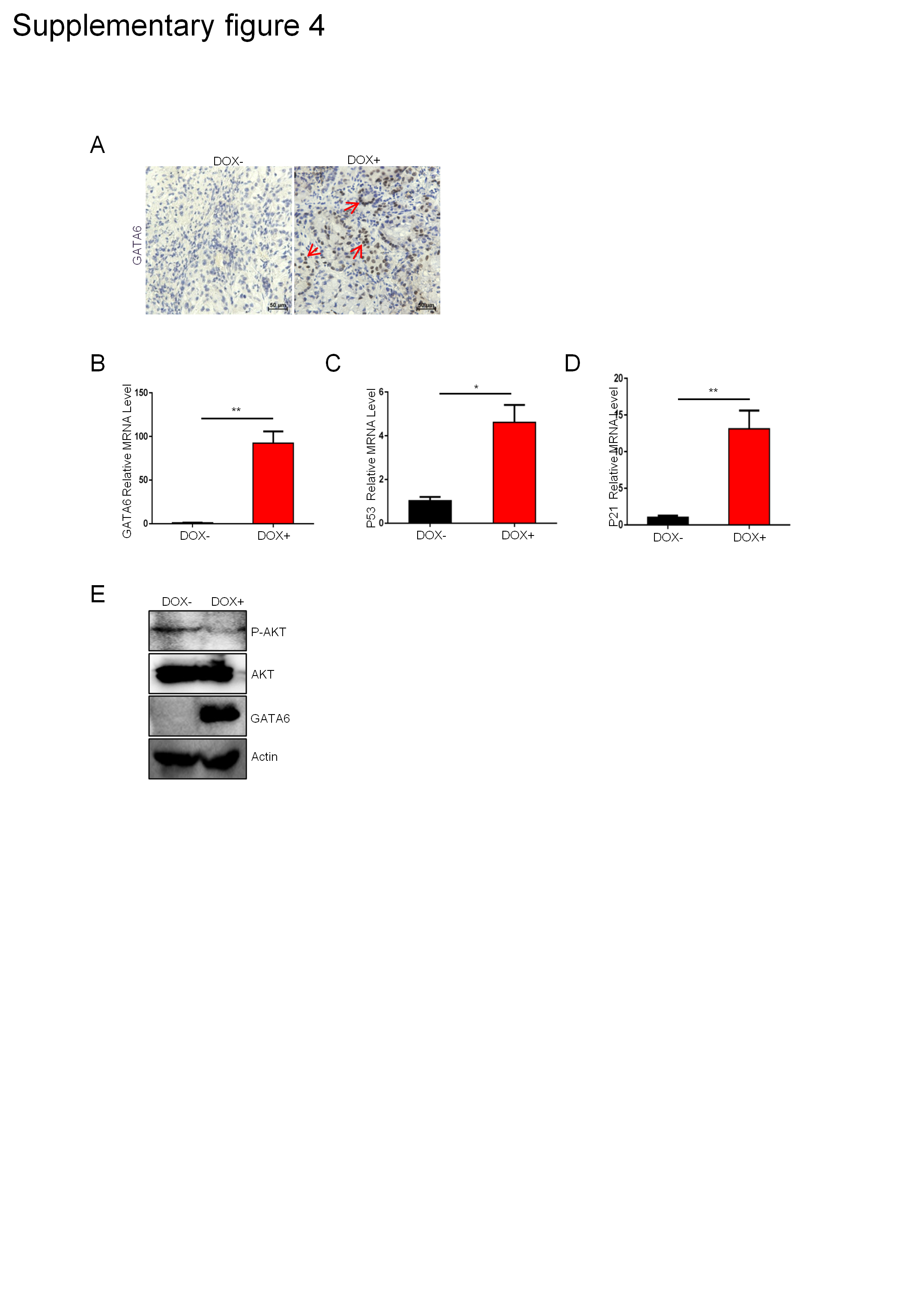

Supplement: Supplementary Figure 4 — (A) Nude mice were inoculated with 5 × 10^6^ A549i cells (harboring DOX inducible expression of GATA6-FLAG), and treated with DOX-containing or control diet for 28 days when tumors reached a volume of 100 mm3. Tumor xenografts were harvested and stained with FLAG-antibody. Tumor cells with heavy nuclear staining of GATA6 were highlighted with arrow heads. (B) qRT-PCR analysis of GATA6 mRNA level in xenografted tumors. (C) qRT-PCR analysis of p53 mRNA level in xenografted tumors. (D) qRT-PCR analysis of p21 mRNA level in xenografted tumors. (E) Western blot analysis of P-AKT, AKT and GATA6 expression in xenografted tumors. Data are representative of three independent experiments, and were analyzed by unpaired t-test Error bars denote SEM. *P < 0.05 and **P < 0.01. [file Image_4.TIF]
